# Developments in gastrointestinal organoid cultures to recapitulate tissue environments

**DOI:** 10.3389/fbioe.2025.1521044

**Published:** 2025-04-17

**Authors:** Madeline R. Kuhn, Emma A. Wolcott, Ellen M. Langer

**Affiliations:** ^1^ Cancer Early Detection Advanced Research Center, Knight Cancer Institute, Oregon Health and Science University, Portland, OR, United States; ^2^ Division of Oncological Sciences, Oregon Health and Science University, Portland, OR, United States; ^3^ Brenden-Colson Center for Pancreatic Care, Oregon Health and Science University, Portland, OR, United States

**Keywords:** organoids, matrix, growth factor signaling, mechanotransduction, *in vitro* models

## Abstract

Culture platforms that closely mimic the spatial architecture, cellular diversity, and extracellular matrix composition of native tissues can serve as invaluable tools for a range of scientific discovery and biomedical applications. Organoids have emerged as a promising alternative to both traditional 2D cell culture and animal models, offering a physiologically relevant 3D culture system for studying human cell biology. Organoids provide a manipulable platform to investigate organ development and function as well as to model patient-specific phenotypes. This mini review examines various methods used for culturing organoids to model normal and disease conditions in gastrointestinal tissues. We focus on how the matrix composition and media formulations can impact cell signaling, altering the baseline cellular phenotypes as well as response to perturbations. We discuss future directions for optimizing organoid culture conditions to improve basic and translational potential.

## Introduction

In the past 2 decades, organoids have greatly expanded our ability to experimentally model gastrointestinal (GI) physiology and pathology. Early studies of two-dimensional cell cultures provided a simple and cost-effective way to study fundamental biology of cells in the GI system and to test phenotypic responses to altered genetics or extrinsic stimuli. However, many GI cells can quickly lose function when cultured in 2D (Elaut et al., 2025; Houbracken et al., 2011). In addition, 2D cultures do not recapitulate the structure and physiology of the native tissues or their microenvironment, leading to an incomplete understanding of the cells and organs in this system. Mouse models, by nature, incorporate this complexity and have been instrumental in advancing understanding of GI organ development, normal homeostasis, and disease. The ability to drive spontaneous pathologies through genetic engineering or inducing injury or inflammation has provided *in vivo* platforms for understanding disease mechanisms and testing therapeutics (Baydi et al., 2021; Klauss et al., 2018; Hayakawa et al., 2013). Mouse models, however, can take a long time to generate, are low throughput, and fail to recapitulate the complexity and heterogeneity found in the patient population at large.

Organoids begin to bridge the gap between cell culture and animal models by mimicking key functional, structural, and biological complexities of organs. Organoid cultures are derived from adult stem cells (ASC) or pluripotent stem cells (PSC) that are grown in a 3D matrix with specific factors that provide mechano-chemical cues. These signals support self-organization of complex structures (Sato et al., 2009; Clevers, 2016; McCracken et al., 2011). The development of organoid cultures has enabled long-term growth of cells from many tissues of the GI system including the intestines, colon, stomach, pancreas, liver, esophagus, and salivary glands (Sato et al., 2009; DeWard et al., 2014; Yoon et al., 2022; Bartfeld et al., 2015; Crespo et al., 2017; Guan et al., 2017; Huang et al., 2021). The function of these models has been demonstrated in normal and disease conditions. For example, heterogeneous salivary gland organoids were shown to display swelling morphology and calcium influx following treatment with neurotransmitters (Yoon et al., 2022) and colon organoids respond to spatiotemporal induction of tumor-associated genetic alterations with increased proliferation and plasticity as they undergo tumorigenesis (Lorenzo-Martin et al., 2024). Moreover, multiple studies comparing global gene expression have now shown organoids to mimic transcriptional profiles of native tissues (Yoon et al., 2022; Paul et al., 2025; Raghavan et al., 2021; Peng et al., 2018; Fujii et al., 2018; Cherubini et al., 2024; Mead et al., 2018).

By mimicking the critical components and function of complex gastrointestinal organs in an *in vitro* system, organoids provide a robust experimental platform to investigate mechanisms of development and disease. Organoids allow for rapid manipulation to mimic genetic alterations or extrinsic stress (Huang et al., 2021; Lorenzo-Martin et al., 2024; Li et al., 2014; Mitrofanova et al., 2024; Farin et al., 2014). In addition, they are amenable to high throughput applications such as drug screening (Du et al., 2020; Mead et al., 2022; Hirt et al., 2022). Finally, they enable modeling patient-to patient heterogeneity and can maintain patient specificity in terms of treatment response, allowing them to serve as models for personalized medicine approaches (Papargyriou et al., 2024; Ooft et al., 2019; Zhao et al., 2024; Demyan et al., 2022; Vlachogiannis et al., 2018). The current field of organoids is quickly evolving, with organoids being incorporated into more complex platforms including co-cultures with stromal and immune components (Saheli et al., 2018; Min et al., 2020; Recaldin et al., 2024), organ-on-a-chip devices (Ballerini et al., 2025; Skardal et al., 2015; Zhang et al., 2017) and bioprinted models (Bernal et al., 2022; Wang et al., 2024). As a new generation of models is further developed and widely adopted, a continued consideration of the variety of mechano-chemical signals provided by culture conditions and how they impact phenotype and function is important.

**FIGURE 1:**
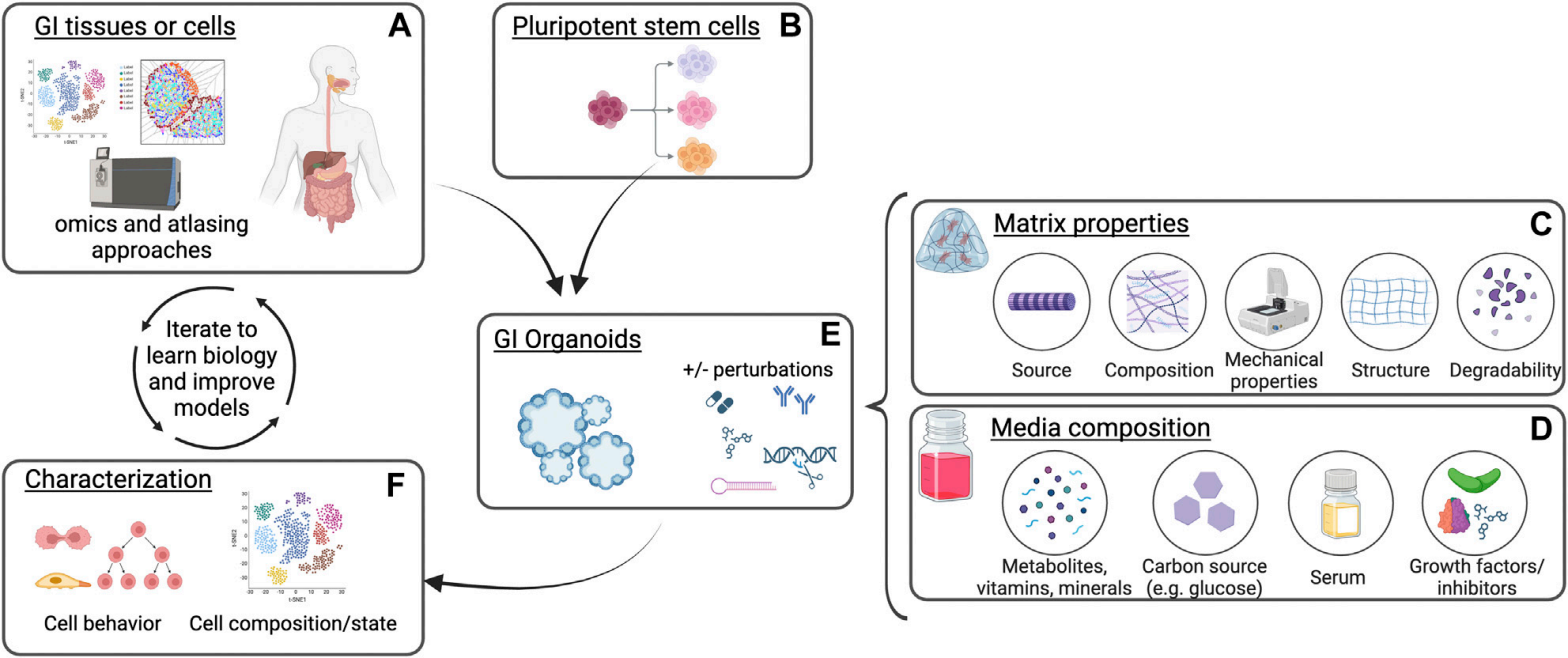
Schematic of gastrointestinal organoid culture and analysis. Gastrointestinal (GI) tissues or cells **(A)** or differentiated pluripotent stem cells **(B)** are plated into extracellular matrix in the presence of culture media. Distinct matrix properties **(C)** and media composition **(D)** can affect organoid establishment, growth, and phenotypes in response to perturbations **(E)**. Characterization of organoid cell states and behaviors in these cultures following modification of culture conditions **(F)** can be compared to native tissues and further optimized to understand biological mechanisms as well as improve the model platform. Created with BioRender.com.

Since the development of the first organoids, several methods for culturing GI organoids have been tested. The nutrients, growth factors, matrix components, and physical cues, such as stiffness, that cells experience influence their signaling pathway usage and phenotype at baseline. They also impact how cells respond to additional perturbations or stress (see Figure 1). Optimizing culture conditions to recapitulate innate biology of tissues can help ensure organoid phenotypes mimic *in vivo* cellular response to perturbations while reductionist models can uncover mechanistic insights into cell and tissue function. In this mini review, we will outline many matrix and media compositions that have been used for culturing organoids of the gastrointestinal system. We will highlight the progress that has been made thus far in creating physiologically relevant environments for organoid culture and will discuss future directions in this field.

## Section 1: matrix

The extracellular matrix (ECM) is a crucial component of the tissue microenvironment. Its composition, organization, and mechanical properties affect cell behavior through chemical signaling and mechanotransduction. In experimental systems such as organoids, the matrix can be chosen to either recapitulate the normal environment or to recapitulate specific components. While mimicking the native matrix gives higher fidelity to the *in vivo* response of organoids (Lee et al., 2021), as of now, there are no perfect matrices that fully recapitulate the native environments of gastrointestinal organs. Reductionist models, therefore, serve as powerful tools for understanding the impact of individual elements, which can then be combined to develop an idealized model.

Several types of matrices have been produced and tested for gastrointestinal organoid culture, including basement membrane extract (BME)-based hydrogels, decellularized ECM hydrogels, defined natural protein hydrogels, hydrogels composed of recombinant proteins and peptides, and synthetic polymer hydrogels (Kozlowski et al., 2021). Each of these matrices has advantages and disadvantages, depending on the experimental design. The material source, composition, cost, availability, ease of use, and variability all need to be considered. Many types of matrices allow for some degree of control over elastic and viscoelastic properties, degradability, and composition (Table 1). These controllable matrix properties, through activation of cell surface receptors such as integrins, can influence downstream signaling pathways. Ultimately, these signals impact epigenetic and transcriptional programs and control organoid phenotypes and cellular behavior (Below et al., 2022; Saraswathibhatla et al., 2023). For example, matrix stiffness leads to integrin activation and focal adhesion assembly. This drives activation and nuclear translocation of YAP/TAZ, which mediate transcriptional responses to the mechanical cues (Dasgupta and McCollum, 2019; Jafarinia et al., 2024; Panciera et al., 2017). *In vivo*, gastrointestinal cells in physiological and pathological states experience a wide range of chemical and physical signals from their native ECM. The mechanisms by which distinct components or properties of the matrix impact cellular behavior are incompletely understood. In this section, we discuss recent developments in several types of matrices that impact gastrointestinal organoid culture efficiency or phenotype.

**TABLE 1:** The advantages and disadvantages of types of matrices used for organoid culture and examples of their use in gastrointestinal organoid culture.

Type of matrix	Advantages	Disadvantages	Organoid examples
Basement membrane extract (BME)-based	• inexpensive • commercially available • used in well-developed protocols	• undefined composition • lot-to-lot variation • lack of tunable mechanical properties • lack of cues necessary for growth/differentiation	Intestine (6, 8, 49-52), Liver (13, 53), Pancreas (54-57), Salivary Gland (10), Esophagus (9)
Decellularized ECM matrix	• preserves native chemical cues	• difficult preparation • limited by donor availability • poorly defined composition • lack of tunable mechanical properties • batch-to-batch variability	Liver (33, 61,67), Intestine (61), Pancreas (61, 62, 63), Stomach (61)
Defined natural protein matrix	• inexpensive • commercially available • defined composition • easily modifiable	• structural information is not preserved • reduced chemical cues • lot-to-lot variation	Liver (24), Pancreas (28, 76 77, 87), Intestine (22, 68-72, 78-81)
Recombinant protein and peptide matrix	• precise placement of chemical cues • mechanically tunable • tunable degradation rate	• high cost • possible endotoxin contamination • possible immunogenicity	Pancreas (86, 88, 89), Intestine (85, 92)
Synthetic polymer matrix	• mechanically tunable • chemically tunable • reproducible • tunable degradation rate	• high cost • requires functionalization with cell-binding peptides or presence of feeder cells • cytoxicity concerns	Liver (101, 102), Intestine (93-96, 98), Pancreas (99)

### Basement membrane extracts

Organoids are commonly cultured in BME matrices such as Matrigel, Cultrex, and Geltrex that are derived from the secretion of mouse sarcoma cells (Kleinman and Martin, 2005). The first organoids cultured using Matrigel were murine intestinal stem cells (Sato et al., 2009), and this system has been adapted for other organoid types including colon (Sato et al., 2011), stomach (Stange et al., 2013; Barker et al., 2010), intestine (Dekkers et al., 2013), liver (Huch et al., 2013a), and pancreas (Huch et al., 2013b; Tiriac et al., 2018; Tsai et al., 2018; Wang et al., 2017; Seino et al., 2018; Lumibao et al., 2024). Basement membrane extracts are versatile, affordable, and readily available, which allows many research groups to easily incorporate organoids into their work, including using organoids in high throughput screens (Du et al., 2020). BME hydrogels, however, have an undefined nature and large batch-to-batch variation. Mechanical properties, including elastic modulus, pore size, stress relaxation, and creep are difficult to separate from chemical cues in these matrices, and the mechanical properties are heterogeneous within each sample (Kozlowski et al., 2021). Commercially available basement membrane extracts are also derived from sarcoma cells and therefore contain growth factors and other chemical cues that are not present in normal cell matrix, so the composition may not be suitable for normal organoid culture (Vukicevic et al., 1992). Due to these limitations, culturing organoids in other matrix types may provide more accurate and consistent results.

### Decellularized ECM matrices

Hydrogels derived from decellularized ECM have been shown to be viable alternatives to basement membrane extracts (Giobbe et al., 2019; Sackett et al., 2018; Chaimov et al., 2017; Gaetani et al., 2018). While BMEs are also ECM-derived, they only contain basement membrane-specific proteins and lack the wider range of proteins found in decellularized tissues. A major benefit of non-BME hydrogels derived from decellularized ECM is the retention of matrix components and chemical cues found in native tissues, including structural proteins, proteoglycans, and glycosaminoglycans (GAGs), which allow researchers to model how the cell’s complex native environment affects signaling and phenotypes. Decellularized ECM hydrogels are produced by treating with detergents to remove cells and then solubilizing the matrix, causing fibers to form into a gel (Saldin et al., 2016). The choice of detergent greatly affects the composition of the final hydrogel, with common detergents including sodium deoxycholate (Giobbe et al., 2019; Sackett et al., 2018), Triton X-100 (Saheli et al., 2018; Chaimov et al., 2017; Gaetani et al., 2018), and sodium dodecyl sulfate (SDS) (Saheli et al., 2018; Gaetani et al., 2018). Using a less harsh detergent, such as Triton X-100, and treating with protease inhibitors can help retain basement membrane and matricellular proteins (Gaetani et al., 2018). Additionally, to address the challenge of incomplete removal lipids from the human pancreas, which contains a higher lipid content than animal pancreata, Dutton Sackett et al. demonstrated a method for decellularizing and delipidizing by performing homogenization prior to sodium deoxycholate treatment (Sackett et al., 2018).

Several groups have shown decellularized ECM matrices to perform equal to or better than Matrigel or collagen in terms of supporting organoid growth and function. Giobbe et al. created a hydrogel using decellularized porcine intestinal tissue that supported human gastric, small intestinal, liver ductal, fetal hepatocyte, and pancreatic organoids. Compared to culture in Matrigel, organoids cultured in the decellularized ECM hydrogels had more similar morphologies and expression profiles to the tissues of origin (Giobbe et al., 2019). Lewis et al. showed that hydrogels derived from porcine liver could support the formation of complex branching biliary networks from cholangiocytes, which was not possible using collagen I hydrogels or Matrigel (Lewis et al., 2018). Saheli et al. co-cultured human hepatocarcinoma cells, bone marrow derived mesenchymal stem cells, and human umbilical cord vein endothelial cells in decellularized ovine liver hydrogel and found these heterotypic liver organoids to have significantly more albumin and alpha-1 antitrypsin secretion, urea production, and CP3A4 enzyme activity as compared to organoids cultured in collagen (Saheli et al., 2018).

Preservation of matrix components and chemical cues of the native environment in decellularized ECM hydrogels supports complex and functional organoid models. However, there are also challenges to using decellularized ECM hydrogels including difficult preparation, lack of precise compositional definition, lack of control over physical properties, and large batch-to-batch variability. Additionally, the quantity of ECM that can be prepared is limited by the availability of starting material, which can be a barrier to scaling up this technology.

### Defined natural protein matrices

Hydrogels with defined protein composition can be formed using individual or combinations of polymers derived from natural sources. Compared to other types of hydrogels, these natural protein matrices have low toxicity and are inexpensive and easily modifiable. There are several well-developed protocols that successfully utilize natural protein matrices such as alginate, fibrin, chitosan, cellulose, gelatin, hyaluron (hyaluronic acid), or collagen for organoid culture (Broguiere et al., 2018; Capeling et al., 2019; Jabaji et al., 2013; Tong et al., 2018; Jabaji et al., 2014; Ng et al., 2019). Protein matrices derived from plants, such as alginate, cellulose, and chitosan, are biocompatible but have no cell adhesion cues. They can be used to create minimally supportive hydrogels or modified with adhesion cues (Capeling et al., 2019; Curvello et al., 2021a). In contrast, hydrogels composed of ECM proteins such as collagen, fibrin, and laminin have inherent adhesion molecules and have been shown to support long-term expansion of murine and human intestinal stem cell organoids equally as well as, or better than, Matrigel in terms of organoid growth and differentiation (Broguiere et al., 2018; Jabaji et al., 2013; Tong et al., 2018). For example, collagen matrices allowed differentiated intestinal epithelial organoids to form enteroid structures and sheet-like growth (Jabaji et al., 2014).

Natural protein matrices have been used in different types of organoid culture, including hydrogel capsules (Liu et al., 2020) and floating gels (Papargyriou et al., 2024; Randriamanantsoa et al., 2022). The floating gels allow organoids to take on different morphologies, such as hollow tubes (Sachs et al., 2017) or branched structures (Papargyriou et al., 2024; Randriamanantsoa et al., 2022). Papargiryriou et al. showed that these branched organoid models could recapitulate phenotypic and transcriptional heterogeneity of mouse and human pancreatic cancer, and that the branching required canonical TGFβ signaling (Papargyriou et al., 2024). Defined natural protein matrices are also compatible with the co-culture of multiple cell types. This has been demonstrated in air-liquid interface cultures that support long-term growth of heterotypic GI organoids containing epithelial and mesenchymal cells (Li et al., 2014; Ootani et al., 2009; Katano et al., 2013) and in co-cultures of pancreatic α- and β-like cells that exhibited glucose-stimulated insulin secretion (Liu et al., 2020).

While defined natural protein matrices are widely used and have been shown to support a variety of organoid cultures, they can exhibit lot-to-lot variation due to their animal origins and it is difficult to tune their mechanical properties without influencing the matrix composition. For example, a high concentration of collagen would be required to reach the stiffness of *in vivo* organs or tissues. To address this, several groups have blended collagen with inert co-polymers such as nanocellulose (Curvello et al., 2021b), alginate (Branco da Cunha et al., 2014), and chitosan (Wang and Stegemann, 2010), which allows hydrogels with a lower collagen concentration to achieve high stiffness while still providing adhesive sites for cell. Altered matrix properties can also be achieved by engineering a natural protein matrix to contain peptides for adhesion or crosslinking (Cruz-Acuña et al., 2023) or by adding recombinant proteins into a hydrogel with defined natural proteins (Hunt et al., 2021; LeSavage et al., 2024) as is discussed in the section below.

### Recombinant protein and peptide matrices

Recombinant proteins and peptides are produced by genetically engineered organisms. Compared to defined natural proteins, recombinant proteins and peptides have the risk of potential endotoxin contamination, but they allow for greater control over mechanical and chemical properties. They can be designed to incorporate precise placement of chemical cues or cell-binding domains and can have tunable mechanical properties and degradation rates. They can, therefore, be used to independently study the effects of cell adhesion and matrix stiffness on organoid formation efficiency and phenotype. The Ku lab developed a hydrogel called aECM-lam that was composed of recombinant elastin-like polypeptide and that incorporated a functional, β1 integrin-binding, IKVAV (Ile-Lys-Val-Ala-Val) sequence derived from laminin. This aECM-lam hydrogel was shown to induce differentiation of CD133^high^;CD71^low^ pancreatic progenitor cells into endocrine and acinar lineages (Jin et al., 2013; Ghazalli et al., 2015; Jin et al., 2016), consistent with published roles for β1 integrin in endocrine differentiation and β-cell proliferation and survival (Pinkse et al., 2006; Diaferia et al., 2013). Similar assays with distinct modified recombinant proteins could further delineate mechanisms of how activation of specific integrins and their downstream signaling drive differentiation and cell behavior. To test the relative contributions of adhesiveness and stiffness, the Heilshorn lab and colleagues used a recombinantly engineered ECM that combines cell-adhesive RGD domains with an elastin-like structural domain that permits differential crosslinking to give variable stiffness. Matrices with decreased stiffness and higher RGD concentrations enhanced intestinal organoid formation. Additionally, organoids cultured in stiffer matrices increased production of matrix metalloproteinases, suggesting that these cells adapt to be able to remodel the matrix into a more compliant environment favorable for their continued growth and development (DiMarco et al., 2015).

Combining recombinant proteins with defined natural proteins can also be used to create matrices with tunable stiffness, stress-relaxation rate, and integrin-ligand concentration, enabling fundamental studies of organoid-matrix interactions. A 3D matrix composed of hyaluronic acid and recombinant elastin-like protein (HELP) enabled formation, differentiation, and passaging of primary human epithelial intestinal organoids (Hunt et al., 2021). Culturing patient-derived pancreatic cancer organoids in HELP matrices showed that high-stiffness drives a reversible resistance to chemotherapy through increased expression of the ATP-binding cassette (ABC) family of drug efflux transporters. The ability to adjust individual components of the matrix also allowed the authors to determine that hyaluronic acid–CD44 signaling, and not integrin signaling, was essential for this stiffness mediated phenotype (LeSavage et al., 2024).

### Synthetic polymer matrices

Hydrogels composed of synthetic polymers such as polyacrylic acid, polyvinyl alcohol, and polyethylene glycol (PEG) are well-defined, highly reproducible, and easily modifiable. Using different methods of production, some of the heterogeneity found in native organs can be recapitulated, including differences in adhesivity, stiffness, viscoelasticity, composition, erosion, and porosity on organoid behavior. Synthetic polymer hydrogels require functionalization with cell-binding peptides or the presence of feeder cells to support organoid culture. These cell-binding peptides can include GFOGER (collagen-I-derived), RGD (fibronectin-derived), lam-5 (laminin-derived), AG73 or IKVAV (both laminin α1 chain-derived), or basement membrane-binding peptides (Below et al., 2022; Hernandez-Gordillo et al., 2020; Gjorevski et al., 2016; Cruz-Acuña et al., 2017; Cruz-Acuña et al., 2018; Valdez et al., 2017). In addition to being designed with specific adhesive domains, the pore size and stiffness of synthetic matrices can be controlled by varying the polymer density, macromer size, and number of reactive arms in the macromer (Mulero-Russe and García, 2024). While the basic materials of these hydrogels, such as PEG and PLGA are inexpensive, the custom-made cell-binding peptides that are required for organoid growth can be costly, and the design and use of synthetic matrices can require materials science expertise.

The defined composition of PEG hydrogels allows for the identification of specific factors that affect organoid formation and behavior. Hernandez-Gordillo et al. compared growth of human duodenal and colon enteroids and endometrial organoids in PEG hydrogels functionalized with either collagen I- or fibronectin-derived peptides. They found that the collagen I-derived GFOGER sequence that binds α2β1 integrin was a critical factor for organoid formation (Hernandez-Gordillo et al., 2020). Control over the composition of hydrogels can also enable differentiation into functional organoids. The chemical structure of Amikagel, a polymerized amikacin hydrate with polyethylene glycol diglycidyl ether, allowed for spontaneous heterotypic organoid formation from co-cultured human embryonic stem cell-derived pancreatic progenitor cells and human umbilical vein endothelial cells. These heterotypic cultures plated in Amikagel had increased expression of C-peptide and insulin and showed a glucose-stimulated increase in insulin (Candiello et al., 2018).

Synthetic polymer matrices, like recombinant peptide matrices, can be used to independently test effects of the chemical composition and adhesion cues as well as mechanical properties such as elastic and viscoelastic modulus, polymer density, and pore size. Pérez-González et al. cultured mouse intestinal organoids in micropatterned polyacrylamide hydrogels coated with collagen and laminin and demonstrated that hydrogel stiffness affects epithelial compartmentalization, crypt folding and collective cell migration (Pérez-González et al., 2021). Gjorevski et al. showed that separate stages of intestinal organoid formation require different stiffnesses and adhesion sites. High matrix stiffness enhanced expansion of intestinal stem cells through YAP-dependent mechanotransduction, while a soft matrix and laminin adhesion was required for differentiation and organoid formation. They modeled a dynamic matrix using a hydrogel with a hydrolytically degradable polymer backbone which allowed gels to soften over time and suggest that synthetic matrices may need to be dynamic in order to recapitulate the native environment (Gjorevski et al., 2016). Other groups have also developed synthetic polymer matrices that can be degraded either by external factors such as light, allowing for precise control over stiffness in different areas of the matrix (Gjorevski et al., 2016), or by factors produced by the embedded cells, allowing for remodeling of the matrix over time (Lee et al., 2017).

Cruz-Acuna et al. tested the independent effects of biochemical composition and mechanical properties on human intestinal organoid formation by creating PEG hydrogels functionalized with adhesive peptides whose stiffness could be changed by changing polymer density. They changed both the type and density of the peptides and showed that this affected organoid development (Cruz-Acuña et al., 2017; Cruz-Acuña et al., 2018). Below et al. developed PEG hydrogels that incorporated collagen-based, fibronectin-based, and basement membrane binding motifs and whose elastic modulus could be tuned by changing the molar ratio of cross-linking peptide. This approach showed that a combination of all three adhesion motifs best supported organoid growth and demonstrated the ability of their synthetic gels to capture the full stiffness range of murine and human pancreatic cancers (Below et al., 2022). Finally, pore size was shown to be an important mechanical property for organoid culture, as it was found that inverted crystal colloidal scaffolds coated with collagen with a pore diameter of 140 µm promoted hepatic differentiation of human adipose-derived mesenchymal stem cells or induced pluripotent stem cells (iPSCs)-derived progenitors (Wang et al., 2016; Ng et al., 2018).

Altogether, there are many options for complex or reductionist matrices for organoid cultures. The different types of matrices have different mechanical and chemical properties, some of which can be manipulated. These factors, along with cost, availability, and ease of use, all may need to be considered (Table 1) as one or more matrix is chosen to address specific biological questions.

## Section 2: media

As discussed above, the matrix composition and mechanical properties play key roles in the formation and organization of organoids and can be altered to drive distinct cell states or to recapitulate dynamic environments. Historically, media was optimized to maintain and propagate tissue cultures, but media composition can mimic developmental signals and niche ligands to serve as a powerful tool to impact cell states and behaviors. In this section, we discuss how media components have been optimized to drive development and differentiation of heterogeneous cultures as well as how they contribute to selection and drug-responses of patient-derived organoids.

Basic nutrients including amino acids, vitamins, salts, metabolites, and proteins are provided to organoid cultures with a base media, often Advanced DMEM/F12. Supplementation with additional factors including antioxidants, such as N-acetylcysteine, vitamins, such as nicotinamide, hormones, such as Gastrin, and a variety of growth factors and/or signaling pathway inhibitors supports the growth of a range of organoid types. For reproducibility, as well as for potential clinical translation, there has been effort toward ensuring that components added to the media are well defined. Serum, for example, can be replaced with defined factors to promote organoid growth including the Wnt agonist R-spondin-1 (RSPO1), epidermal growth factor (EGF), and Noggin (Sato et al., 2009). Defined medias are considerably more expensive than some alternatives including conditioned media from growth factor producing cells. However, leftover serum and other undefined factors in conditioned media have the potential to introduce lot-to-lot variability affecting experimental outcomes. A recent assessment of conditioned media from the commonly used L-WRN producer cell line across five laboratories at three institutions found highly replicable Wnt3a, R-spondin3, and Noggin growth factor activity across all groups (VanDussen et al., 2019). In general, however, similar to control of the matrix components discussed above, reproducibility of organoid experiments can be enhanced with defined media components.

Signaling pathway activators and inhibitors that are critical for the development, differentiation, or maintenance of organ-specific cell types can be added to organoid cultures to drive PSCs or ASCs toward mature fates (Clevers, 2016; Lancaster and Knoblich, 2014). In early organoid work, Sato et al. showed that intestinal ASCs required RSPO1, EGF, and Noggin for proliferation and successful passaging as organoids (Sato et al., 2009). Spence et al. drove human PSCs toward hindgut cell fates with Activin A, Wnt, and fibroblast growth factor (FGF) treatments, and then cultured them in Matrigel with RSPO1, EGF, and Noggin to develop intestinal organoids (Spence et al., 2011). Huch et al. cultured liver stem cells with RSPO1, EGF, FGF10, hepatocyte growth factor (HGF), and nicotinamide to develop liver progenitor cultures. Additional inhibition of Notch and transforming growth factor beta (TGFβ) signaling drove expression of mature hepatocyte markers and some hepatocyte function (Huch et al., 2013a). In similar approaches, recapitulating developmental signaling pathways or niche ligands has allowed development of distinct cell fates or the balanced differentiation of heterogenous cultures. Huang et al. used multiple signaling ligands and inhibitors to induce pancreatic progenitors to differentiate to either acinar or ductal fates. They further built on this system, showing lineage-specific responses to the expression of distinct oncogenes GNAS^R201C^ and KRAS^G12D^ as well as differential transcriptional and morphological responses to TGFβ signaling in KRAS^G12D^-expressing acinar *versus* ductal organoids (Huang et al., 2021). Yoon et al. used a variety of growth factors and inhibitors to refine culture conditions for salivary organoids to promote long-term growth as well as phenotypic and functional heterogeneity of salivary glands (Yoon et al., 2022). Yang et al. optimized niche signals to drive a balance in intestinal stem cell proliferation and differentiation in their organoid cultures and showed that manipulation of specific signaling pathways could shift that balance and drive specific intestinal fates (Yang et al., 2025). Finally, Mead et al. used high throughput screening on a miniaturized organoid platform to identify new regulators of intestinal stem cell differentiation (Mead et al., 2022).

As organoid research has expanded to model patient-derived tissues from specific pathologies, including cancer, there has been recognition that specific media components contribute to the efficiency of organoid generation from a heterogeneous patient population and to the selection of subpopulations of patient-derived cells. Seino et al. developed a library of 39 patient-derived organoids from pancreatic cancer and identified three functional subtypes of these organoids based on their dependence on Wnt and R-spondin in the media. They also altered the media composition with EGF depletion, Noggin depletion, or Nutlin3 addition to intentionally select for pancreatic cancer organoids expressing mutant KRAS, mutant SMAD4, or mutant p53, respectively (Seino et al., 2018). Hawkins et al. similarly observed different patient-derived pancreatic cancer organoid lines required distinct concentrations of Wnt. They further showed that this information may be useful in designing combination treatment strategies (Hawkins et al., 2024).

Patient-derived organoids provide an opportunity not only to study the biology underlying tumorigenic phenotypes, but also to test patient-specific responses to therapeutics. Tiriac et al., for example, developed a biobank of 66 patient-derived pancreatic cancer organoids and demonstrated that the drug sensitivity profiles of the organoids reflected the patient response to therapy (Tiriac et al., 2018). Huang et al. leveraged patient-derived xenografts (PDX) of pancreatic cancer to compare the therapeutic sensitivity of the PDX models to that of PDX-derived organoids. They demonstrated that, when grown in Wnt-free media, the organoids retained the tumor differentiation status and histological heterogeneity and were concordant with the PDXs for drug sensitivity (Huang et al., 2020). Hogenson et al. evaluated patient-derived organoids from multiple gastrointestinal cancers for their ability to predict clinical response. They also found that Wnt in the organoid media impacted transcriptional signatures and drug sensitivity, with the media lacking Wnt giving better concordance with patient responses (Hogenson et al., 2022). These studies indicate that factors in the media can critically affect the interpretation of organoid response to specific stressors. Raghavan et al. analyzed matched pancreatic cancer patient samples and organoid models to investigate how microenvironmental signals present at distinct sites or associated with distinct molecular subtypes could control organoid state or drug response. They found that organoid cultures supplemented with TGFβ exhibited plasticity toward a basal-like phenotype and had therapeutic responses distinct from organoids with classical gene expression. In addition, treatment of the organoids with IFNγ to model signaling from CD8^+^ T cells, was found to increase IFN response gene signatures and drive an intermediate state expressing both basal and classical programs (Raghavan et al., 2021).

## Conclusion and future directions

Together, the properties of the matrix and media in which organoids are cultured can alter the efficiency of organoid generation, affect signaling pathways leading to phenotypic changes, and alter the responses to perturbation. Many recent studies of patient-derived tumor organoids have focused on the presence of Wnt ligands in the media, finding Wnt signaling to be a major player in driving organoid selection, heterogeneity and drug sensitivity (Huang et al., 2020; Hogenson et al., 2022). TGFβ was found to alter organoid subtype, which also impacted drug sensitivity (Raghavan et al., 2021). Future work using high throughput screens to test responses to exogenous ligands, inhibitors, or drugs that impact specific signaling pathways (Mead et al., 2022; Hirt et al., 2022) can be leveraged to continue to uncover mechanisms driving distinct organoid phenotypes or vulnerabilities. As integrins and other receptors regulating mechanotransduction cooperate with many growth factor receptors, including those in the Wnt pathway (Crampton et al., 2009; Tejeda-Muñoz et al., 2022; Cooper and Giancotti, 2019; Sarker et al., 2020; Li et al., 2023), it will also be of interest in future work to study these signals in combination. Through adaptation of the media along with the matrix, organoid studies can address how specific signaling pathways converge to contribute to lineage commitment and progression, function, and response to perturbations.

The matrix and media can be chosen to recapitulate the native environment or can be designed in a reductionist approach to understand specific aspects of cellular responses to the environment. Matrices that support organoid growth, for example, can range from inert gels with a single adhesive site to the complex decellularized ECM from the tissue of interest. Proteomics analyses on ECM-derived matrices has been used to compare hydrogels to native matrices (Giobbe et al., 2019). Proteomics studies have also enabled the design of manipulable gels in which specific components of the native environment can be modulated to define which matrix proteins or properties control cell fates (Below et al., 2022). Extending these types of studies to additional tissues or disease states will be of interest. Similarly, large scale atlasing of transcriptional profiles in normal and tumor tissues is providing unprecedented information about the ligands, receptors, and signaling pathways that control cell-cell communication in niche environments (Yanai et al., 2024)*.* As these studies suggest additional pathways to control through changes in the media or matrix, single cell transcriptomic approaches can be used to compare organoid cell composition and cell state to that found in normal tissues to validate these efforts (Yoon et al., 2022; Fujii et al., 2018; Cherubini et al., 2024; Mead et al., 2022).

Future work incorporating GI organoids into increasingly complex model platforms will increase understanding of organ development, homeostatic function, and disease. In addition, leveraging patient-derived GI organoids in precision medicine approaches may provide routes for clinical translation. Throughout these studies, systematic perturbation of the matrix and media components will allow for continued interrogation of mechanical and chemical signals regulating gastrointestinal physiology and pathology.

## References

[B1] Ballerini M. Galiè S. Tyagi P. Catozzi C. Raji H. Nabinejad A. (2025). A gut-on-a-chip incorporating human faecal samples and peristalsis predicts responses to immune checkpoint inhibitors for melanoma. Nat. Biomed. Eng., 1–18. 10.1038/s41551-024-01318-z 39939548 PMC12176660

[B2] Barker N. Huch M. Kujala P. van de Wetering M. Snippert H. J. van Es J. H. (2010). Lgr5+ve stem cells drive self-renewal in the stomach and build long-Lived gastric Units *in vitro* . Cell Stem Cell 6 (1), 25–36. 10.1016/j.stem.2009.11.013 20085740

[B3] Barrett A. S. Wither M. J. Hill R. C. Dzieciatkowska M. D’Alessandro A. Reisz J. A. (2017). Hydroxylamine chemical Digestion for Insoluble extracellular matrix Characterization. J. Proteome Res. 16 (11), 4177–4184. 10.1021/acs.jproteome.7b00527 28971683 PMC5802359

[B4] Bartfeld S. Bayram T. van de Wetering M. Huch M. Begthel H. Kujala P. (2015). *In vitro* expansion of human gastric epithelial stem cells and their responses to Bacterial Infection. Gastroenterology 148 (1), 126–136.e6. 10.1053/j.gastro.2014.09.042 25307862 PMC4274199

[B5] Baydi Z. Limami Y. Khalki L. Zaid N. Naya A. Mtairag E. M. (2021). An Update of research animal models of inflammatory Bowel disease. Sci. World J. 2021 (1), 1–12. 10.1155/2021/7479540 PMC8687830 34938152

[B6] Below C. R. Kelly J. Brown A. Humphries J. D. Hutton C. Xu J. (2022). A microenvironment-inspired synthetic three-dimensional model for pancreatic ductal adenocarcinoma organoids. Nat. Mater 21 (1), 110–119. 10.1038/s41563-021-01085-1 34518665 PMC7612137

[B7] Bernal P. N. Bouwmeester M. Madrid-Wolff J. Falandt M. Florczak S. Rodriguez N. G. (2022). Volumetric bioprinting of organoids and Optically tuned hydrogels to build liver-like metabolic Biofactories. Adv. Mater 34 (15), 2110054. 10.1002/adma.202110054 35166410

[B8] Branco da Cunha C. Klumpers D. D. Li W. A. Koshy S. T. Weaver J. C. Chaudhuri O. (2014). Influence of the stiffness of three-dimensional alginate/collagen-I interpenetrating networks on fibroblast biology. Biomaterials 35 (32), 8927–8936. 10.1016/j.biomaterials.2014.06.047 25047628

[B9] Broguiere N. Isenmann L. Hirt C. Ringel T. Placzek S. Cavalli E. (2018). Growth of epithelial organoids in a defined hydrogel. Adv. Mater 30 (43), 1801621. 10.1002/adma.201801621 30203567

[B10] Candiello J. Grandhi T. S. P. Goh S. K. Vaidya V. Lemmon-Kishi M. Eliato K. R. (2018). 3D heterogeneous islet organoid generation from human embryonic stem cells using a novel engineered hydrogel platform. Biomaterials 177, 27–39. 10.1016/j.biomaterials.2018.05.031 29883914

[B11] Capeling M. M. Czerwinski M. Huang S. Tsai Y. H. Wu A. Nagy M. S. (2019). Nonadhesive alginate hydrogels support growth of pluripotent stem cell-derived intestinal organoids. Stem Cell Rep. 12 (2), 381–394. 10.1016/j.stemcr.2018.12.001 PMC6373433 30612954

[B12] Chaimov D. Baruch L. Krishtul S. Meivar-levy I. Ferber S. Machluf M. (2017). Innovative encapsulation platform based on pancreatic extracellular matrix achieve substantial insulin delivery. J. Control. Release 257, 91–101. 10.1016/j.jconrel.2016.07.045 27476611

[B13] Cherubini A. Rusconi F. Piras R. Wächtershäuser K. N. Dossena M. Barilani M. (2024). Exploring human pancreatic organoid modelling through single-cell RNA sequencing analysis. Commun. Biol. 7 (1), 1–16. 10.1038/s42003-024-07193-3 39558019 PMC11574267

[B14] Clevers H. (2016). Modeling development and disease with organoids. Cell 165 (7), 1586–1597. 10.1016/j.cell.2016.05.082 27315476

[B15] Cooper J. Giancotti F. G. (2019). Integrin signaling in cancer: mechanotransduction, Stemness, epithelial plasticity, and therapeutic resistance. Cancer Cell 35 (3), 347–367. 10.1016/j.ccell.2019.01.007 30889378 PMC6684107

[B16] Crampton S. P. Wu B. Park E. J. Kim J. H. Solomon C. Waterman M. L. (2009). Integration of the β-Catenin-dependent Wnt pathway with integrin signaling through the adaptor molecule Grb2. PLOS ONE 4 (11), e7841. 10.1371/journal.pone.0007841 19924227 PMC2773007

[B17] Crespo M. Vilar E. Tsai S. Y. Chang K. Amin S. Srinivasan T. (2017). Colonic organoids derived from human induced pluripotent stem cells for modeling colorectal cancer and drug testing. Nat. Med. 23 (7), 878–884. 10.1038/nm.4355 28628110 PMC6055224

[B18] Cruz-Acuña R. Kariuki S. W. Sugiura K. Karaiskos S. Plaster E. M. Loebel C. (2023). Engineered hydrogel reveals contribution of matrix mechanics to esophageal adenocarcinoma and identifies matrix-activated therapeutic targets. J. Clin. Invest 133 (23), e168146. 10.1172/jci168146 37788109 PMC10688988

[B19] Cruz-Acuña R. Quirós M. Farkas A. E. Dedhia P. H. Huang S. Siuda D. (2017). Synthetic hydrogels for human intestinal organoid generation and colonic wound repair. Nat. Cell Biol. 19 (11), 1326–1335. 10.1038/ncb3632 29058719 PMC5664213

[B20] Cruz-Acuña R. Quirós M. Huang S. Siuda D. Spence J. R. Nusrat A. (2018). PEG-4MAL hydrogels for human organoid generation, culture, and *in vivo* delivery. Nat. Protoc. 13 (9), 2102–2119. 10.1038/s41596-018-0036-3 30190557 PMC7240347

[B21] Curvello R. Alves D. Abud H. E. Garnier G. (2021b). A thermo-responsive collagen-nanocellulose hydrogel for the growth of intestinal organoids. Mater Sci. Eng. C 124, 112051. 10.1016/j.msec.2021.112051 33947545

[B22] Curvello R. Kerr G. Micati D. J. Chan W. H. Raghuwanshi V. S. Rosenbluh J. (2021a). Engineered Plant-based nanocellulose hydrogel for small intestinal organoid growth. Adv. Sci. 8 (1), 2002135. 10.1002/advs.202002135 PMC7788499 33437574

[B23] Dasgupta I. McCollum D. (2019). Control of cellular responses to mechanical cues through YAP/TAZ regulation. J. Biol. Chem. 294 (46), 17693–17706. 10.1074/jbc.rev119.007963 31594864 PMC6873206

[B24] Dekkers J. F. Wiegerinck C. L. de Jonge H. R. Bronsveld I. Janssens H. M. de Winter-de Groot K. M. (2013). A functional CFTR assay using primary cystic fibrosis intestinal organoids. Nat. Med. 19 (7), 939–945. 10.1038/nm.3201 23727931

[B25] Demyan L. Habowski A. N. Plenker D. King D. A. Standring O. J. Tsang C. (2022). Pancreatic cancer patient-derived organoids can predict response to Neoadjuvant chemotherapy. Ann. Surg. 276 (3), 450–462. 10.1097/sla.0000000000005558 35972511 PMC10202108

[B26] DeWard A. D. Cramer J. Lagasse E. (2014). Cellular heterogeneity in the mouse esophagus Implicates the presence of a Nonquiescent epithelial stem cell population. Cell Rep. 9 (2), 701–711. 10.1016/j.celrep.2014.09.027 25373907 PMC4223874

[B27] Diaferia G. R. Jimenez-Caliani A. J. Ranjitkar P. Yang W. Hardiman G. Rhodes C. J. (2013). β1 integrin is a crucial regulator of pancreatic β-cell expansion. Development 140 (16), 3360–3372. 10.1242/dev.098533 23863477 PMC3737718

[B28] DiMarco R. L. Dewi R. E. Bernal G. Kuo C. Heilshorn S. C. (2015). Protein-engineered scaffolds for *in vitro* 3D culture of primary adult intestinal organoids. Biomater. Sci. 3 (10), 1376–1385. 10.1039/c5bm00108k 26371971 PMC9063856

[B29] Du Y. Li X. Niu Q. Mo X. Qui M. Ma T. (2020). Development of a miniaturized 3D organoid culture platform for ultra-high-throughput screening. J. Mol. Cell Biol. 12 (8), 630–643. 10.1093/jmcb/mjaa036 32678871 PMC7751183

[B30] Elaut G. Henkens T. Papeleu P. Snykers S. Vinken M. Vanhaecke T. (2025). Molecular mechanisms underlying the Dedifferentiation Process of Isolated hepatocytes and their cultures. Available online at: https://www.eurekaselect.com/article/1400. 10.2174/138920006778017759 16918317

[B31] Farin H. F. Karthaus W. R. Kujala P. Rakhshandehroo M. Schwank G. Vries R. G. J. (2014). Paneth cell extrusion and release of antimicrobial products is directly controlled by immune cell–derived IFN-γ. J. Exp. Med. 211 (7), 1393–1405. 10.1084/jem.20130753 24980747 PMC4076587

[B32] Fujii M. Matano M. Toshimitsu K. Takano A. Mikami Y. Nishikori S. (2018). Human intestinal organoids maintain self-renewal Capacity and cellular diversity in niche-inspired culture condition. Cell Stem Cell 23 (6), 787–793.e6. 10.1016/j.stem.2018.11.016 30526881

[B33] Gaetani R. Aude S. DeMaddalena L. L. Strassle H. Dzieciatkowska M. Wortham M. (2018). Evaluation of different Decellularization protocols on the generation of pancreas-derived hydrogels. Tissue Eng. Part C Methods 24 (12), 697–708. 10.1089/ten.tec.2018.0180 30398401 PMC6306687

[B34] Ghazalli N. Mahdavi A. Feng T. Jin L. Kozlowski M. T. Hsu J. (2015). Postnatal pancreas of Mice contains tripotent progenitors capable of giving Rise to duct, acinar, and endocrine cells *in vitro* . Stem Cells Dev. 24 (17), 1995–2008. 10.1089/scd.2015.0007 25941840 PMC4545527

[B35] Giobbe G. G. Crowley C. Luni C. Campinoti S. Khedr M. Kretzschmar K. (2019). Extracellular matrix hydrogel derived from decellularized tissues enables endodermal organoid culture. Nat. Commun. 10 (1), 5658. 10.1038/s41467-019-13605-4 31827102 PMC6906306

[B36] Gjorevski N. Sachs N. Manfrin A. Giger S. Bragina M. E. Ordóñez-Morán P. (2016). Designer matrices for intestinal stem cell and organoid culture. Nature 539 (7630), 560–564. 10.1038/nature20168 27851739

[B37] Guan Y. Xu D. Garfin P. M. Ehmer U. Hurwitz M. Enns G. (2017). Human hepatic organoids for the analysis of human genetic diseases. JCI Insight 2 (17), e94954. 10.1172/jci.insight.94954 28878125 PMC5621886

[B38] Hawkins H. J. Yacob B. W. Brown M. E. Goldstein B. R. Arcaroli J. J. Bagby S. M. (2024). Examination of Wnt signaling as a therapeutic target for pancreatic ductal adenocarcinoma (PDAC) using a pancreatic tumor organoid library (PTOL). PLOS ONE 19 (4), e0298808. 10.1371/journal.pone.0298808 38598488 PMC11006186

[B39] Hayakawa Y. Fox J. G. Gonda T. Worthley D. L. Muthupalani S. Wang T. C. (2013). Mouse models of gastric cancer. Cancers 5 (1), 92–130. 10.3390/cancers5010092 24216700 PMC3730302

[B40] Hernandez-Gordillo V. Kassis T. Lampejo A. Choi G. Gamboa M. E. Gnecco J. S. (2020). Fully synthetic matrices for *in vitro* culture of primary human intestinal enteroids and endometrial organoids. Biomaterials 254, 120125. 10.1016/j.biomaterials.2020.120125 32502894 PMC8005336

[B41] Hirt C. K. Booij T. H. Grob L. Simmler P. Toussaint N. C. Keller D. (2022). Drug screening and genome editing in human pancreatic cancer organoids identifies drug-gene interactions and candidates for off-label therapy. Cell Genomics 2 (2), 100095. 10.1016/j.xgen.2022.100095 35187519 PMC7612395

[B42] Hogenson T. L. Xie H. Phillips W. J. Toruner M. D. Li J. J. Horn I. P. (2022). Culture media composition influences patient-derived organoid ability to predict therapeutic responses in gastrointestinal cancers. JCI Insight 7 (22), e158060. 10.1172/jci.insight.158060 36256477 PMC9746806

[B43] Houbracken I. de Waele E. Lardon J. Ling Z. Heimberg H. Rooman I. (2011). Lineage Tracing Evidence for Transdifferentiation of acinar to duct cells and plasticity of human pancreas. Gastroenterology 141 (2), 731–741.e4. 10.1053/j.gastro.2011.04.050 21703267

[B44] Huang L. Bockorny B. Paul I. Akshinthala D. Frappart P. O. Gandarilla O. (2020). PDX-derived organoids model *in vivo* drug response and secrete biomarkers. JCI Insight 5 (21), e135544. 10.1172/jci.insight.135544 32990680 PMC7710298

[B45] Huang L. Desai R. Conrad D. N. Leite N. C. Akshinthala D. Lim C. M. (2021). Commitment and oncogene-induced plasticity of human stem cell-derived pancreatic acinar and ductal organoids. Cell Stem Cell 28 (6), 1090–1104.e6. 10.1016/j.stem.2021.03.022 33915081 PMC8202734

[B46] Huch M. Bonfanti P. Boj S. F. Sato T. Loomans C. J. M. van de Wetering M. (2013b). Unlimited *in vitro* expansion of adult bi‐potent pancreas progenitors through the Lgr5/R‐spondin axis. EMBO J. 32 (20), 2708–2721. 10.1038/emboj.2013.204 24045232 PMC3801438

[B47] Huch M. Dorrell C. Boj S. F. van Es J. H. Li V. S. W. van de Wetering M. (2013a). *In vitro* expansion of single Lgr5+ liver stem cells induced by Wnt-driven regeneration. Nature 494 (7436), 247–250. 10.1038/nature11826 23354049 PMC3634804

[B48] Hunt D. R. Klett K. C. Mascharak S. Wang H. Gong D. Lou J. (2021). Engineered matrices enable the culture of human patient-derived intestinal organoids. Adv. Sci. 8 (10), 2004705. 10.1002/advs.202004705 PMC8132048 34026461

[B49] Jabaji Z. Brinkley G. J. Khalil H. A. Sears C. M. Lei N. Y. Lewis M. (2014). Type I collagen as an extracellular matrix for the *in vitro* growth of human small intestinal Epithelium. PLOS ONE 9 (9), e107814. 10.1371/journal.pone.0107814 25222024 PMC4164635

[B50] Jabaji Z. Sears C. M. Brinkley G. J. Lei N. Y. Joshi V. S. Wang J. (2013). Use of collagen gel as an alternative extracellular matrix for the *in vitro* and *in vivo* growth of murine small intestinal Epithelium. Tissue Eng. Part C Methods 19 (12), 961–969. 10.1089/ten.tec.2012.0710 23566043 PMC3833386

[B51] Jafarinia H. Khalilimeybodi A. Barrasa-Fano J. Fraley S. I. Rangamani P. Carlier A. (2024). Insights gained from computational modeling of YAP/TAZ signaling for cellular mechanotransduction. Npj Syst. Biol. Appl. 10 (1), 90–14. 10.1038/s41540-024-00414-9 39147782 PMC11327324

[B52] Jin L. Feng T. Shih H. P. Zerda R. Luo A. Hsu J. (2013). Colony-forming cells in the adult mouse pancreas are expandable in Matrigel and form endocrine/acinar colonies in laminin hydrogel. Proc. Natl. Acad. Sci. 110 (10), 3907–3912. 10.1073/pnas.1301889110 23431132 PMC3593860

[B53] Jin L. Gao D. Feng T. Tremblay J. R. Ghazalli N. Luo A. (2016). Cells with surface expression of CD133highCD71low are enriched for tripotent colony-forming progenitor cells in the adult murine pancreas. Stem Cell Res. 16 (1), 40–53. 10.1016/j.scr.2015.11.015 26691820 PMC4762724

[B54] Katano T. Ootani A. Mizoshita T. Tanida S. Tsukamoto H. Ozeki K. (2013). Establishment of a long-term three-dimensional primary culture of mouse glandular stomach epithelial cells within the stem cell niche. Biochem. Biophys. Res. Commun. 432 (4), 558–563. 10.1016/j.bbrc.2013.02.051 23485463

[B55] Klauss S. Schorn S. Teller S. Steenfadt H. Friess H. Ceyhan G. O. (2018). Genetically induced vs. classical animal models of chronic pancreatitis: a critical comparison. FASEB J. 32 (11), 5778–5792. 10.1096/fj.201800241rr 29863911

[B56] Kleinman H. K. Martin G. R. (2005). Matrigel: basement membrane matrix with biological activity. Semin. Cancer Biol. 15 (5), 378–386. 10.1016/j.semcancer.2005.05.004 15975825

[B57] Kozlowski M. T. Crook C. J. Ku H. T. (2021). Towards organoid culture without Matrigel. Commun. Biol. 4 (1), 1–15. 10.1038/s42003-021-02910-8 34893703 PMC8664924

[B58] Lancaster M. A. Knoblich J. A. (2014). Organogenesis in a dish: modeling development and disease using organoid technologies. Science 345 (6194), 1247125. 10.1126/science.1247125 25035496

[B59] Lee H. J. Mun S. Pham D. M. Kim P. (2021). Extracellular matrix-based hydrogels to Tailoring tumor organoids. ACS Biomater. Sci. Eng. 7 (9), 4128–4135. 10.1021/acsbiomaterials.0c01801 33724792

[B60] Lee H. J. Son M. J. Ahn J. Oh S. J. Lee M. Kim A. (2017). Elasticity-based development of functionally enhanced multicellular 3D liver encapsulated in hybrid hydrogel. Acta Biomater. 64, 67–79. 10.1016/j.actbio.2017.09.041 28966094

[B61] Lee K. Y. Mooney D. J. (2012). Alginate: properties and biomedical applications. Prog. Polym. Sci. 37 (1), 106–126. 10.1016/j.progpolymsci.2011.06.003 22125349 PMC3223967

[B62] LeSavage B. L. Zhang D. Huerta-López C. Gilchrist A. E. Krajina B. A. Karlsson K. (2024). Engineered matrices reveal stiffness-mediated chemoresistance in patient-derived pancreatic cancer organoids. Nat. Mater, 1–12. 10.1038/s41563-024-01908-x 38965405 PMC13098013

[B63] Lewis P. L. Su J. Yan M. Meng F. Glaser S. S. Alpini G. D. (2018). Complex bile duct network formation within liver decellularized extracellular matrix hydrogels. Sci. Rep. 8 (1), 12220. 10.1038/s41598-018-30433-6 30111800 PMC6093899

[B64] Li S. Sampson C. Liu C. Piao H. long Liu H. X. (2023). Integrin signaling in cancer: bidirectional mechanisms and therapeutic opportunities. Cell Commun. Signal 21 (1), 266. 10.1186/s12964-023-01264-4 37770930 PMC10537162

[B65] Li X. Nadauld L. Ootani A. Corney D. C. Pai R. K. Gevaert O. (2014). Oncogenic transformation of diverse gastrointestinal tissues in primary organoid culture. Nat. Med. 20 (7), 769–777. 10.1038/nm.3585 24859528 PMC4087144

[B66] Liu H. Wang Y. Wang H. Zhao M. Tao T. Zhang X. (2020). A Droplet Microfluidic system to Fabricate hybrid capsules enabling stem cell organoid engineering. Adv. Sci. 7 (11), 1903739. 10.1002/advs.201903739 PMC7284190 32537414

[B67] Lorenzo-Martin L. F. Hubscher T. Bowler A. D. Broguiere N. Langer J. Tillard L. (2024). Spatiotemporally resolved colorectal oncogenesis in mini-colons *ex vivo* . Nature 629, 450–457. 10.1038/s41586-024-07330-2 38658753 PMC11078756

[B68] Lumibao J. C. Okhovat S. R. Peck K. L. Lin X. Lande K. Yomtoubian S. (2024). The effect of extracellular matrix on the precision medicine utility of pancreatic cancer patient–derived organoids. JCI Insight 9 (1), e172419. 10.1172/jci.insight.172419 38051586 PMC10906458

[B69] McCracken K. W. Howell J. C. Wells J. M. Spence J. R. (2011). Generating human intestinal tissue from pluripotent stem cells *in vitro* . Nat. Protoc. 6 (12), 1920–1928. 10.1038/nprot.2011.410 22082986 PMC3896236

[B70] Mead B. E. Hattori K. Levy L. Imada S. Goto N. Vukovic M. (2022). Screening for modulators of the cellular composition of gut epithelia via organoid models of intestinal stem cell differentiation. Nat. Biomed. Eng. 6 (4), 476–494. 10.1038/s41551-022-00863-9 35314801 PMC9046079

[B71] Mead B. E. Ordovas-Montanes J. Braun A. P. Levy L. E. Bhargava P. Szucs M. J. (2018). Harnessing single-cell genomics to improve the physiological fidelity of organoid-derived cell types. BMC Biol. 16 (1), 62. 10.1186/s12915-018-0527-2 29871632 PMC5989470

[B72] Min S. Kim S. Cho S. W. (2020). Gastrointestinal tract modeling using organoids engineered with cellular and microbiota niches. Exp. Mol. Med. 52 (2), 227–237. 10.1038/s12276-020-0386-0 32103122 PMC7062772

[B73] Mitrofanova O. Nikolaev M. Xu Q. Broguiere N. Cubela I. Camp J. G. (2024). Bioengineered human colon organoids with *in vivo*-like cellular complexity and function. Cell Stem Cell 31 (8), 1175–1186.e7. 10.1016/j.stem.2024.05.007 38876106

[B74] Mulero-Russe A. García A. J. (2024). Engineered synthetic matrices for human intestinal organoid culture and therapeutic delivery. Adv. Mater 36 (9), 2307678. 10.1002/adma.202307678 PMC10922691 37987171

[B75] Ng S. Tan W. J. Pek M. M. X. Tan M. H. Kurisawa M. (2019). Mechanically and chemically defined hydrogel matrices for patient-derived colorectal tumor organoid culture. Biomaterials 219, 119400. 10.1016/j.biomaterials.2019.119400 31398570

[B76] Ng S. S. Saeb-Parsy K. Blackford S. J. I. Segal J. M. Serra M. P. Horcas-Lopez M. (2018). Human iPS derived progenitors bioengineered into liver organoids using an inverted colloidal crystal poly (ethylene glycol) scaffold. Biomaterials 182, 299–311. 10.1016/j.biomaterials.2018.07.043 30149262 PMC6131727

[B77] Ooft S. N. Weeber F. Dijkstra K. K. McLean C. M. Kaing S. van Werkhoven E. (2019). Patient-derived organoids can predict response to chemotherapy in metastatic colorectal cancer patients. Sci. Transl. Med. 11 (513), eaay2574. 10.1126/scitranslmed.aay2574 31597751

[B78] Ootani A. Li X. Sangiorgi E. Ho Q. T. Ueno H. Toda S. (2009). Sustained *in vitro* intestinal epithelial culture within a Wnt-dependent stem cell niche. Nat. Med. 15 (6), 701–706. 10.1038/nm.1951 19398967 PMC2919216

[B79] Panciera T. Azzolin L. Cordenonsi M. Piccolo S. (2017). Mechanobiology of YAP and TAZ in physiology and disease. Nat. Rev. Mol. Cell Biol. 18 (12), 758–770. 10.1038/nrm.2017.87 28951564 PMC6192510

[B80] Papargyriou A. Najajreh M. Cook D. P. Maurer C. H. Bärthel S. Messal H. A. (2024). Heterogeneity-driven phenotypic plasticity and treatment response in branched-organoid models of pancreatic ductal adenocarcinoma. Nat. Biomed. Eng. 10, 1–29. 10.1038/s41551-024-01273-9 PMC12176653 39658630

[B81] Paul C. D. Yankaskas C. Shahi Thakuri P. Balhouse B. Salen S. Bullock A. (2025). Long-term maintenance of patient-specific characteristics in tumoroids from six cancer indications. Sci. Rep. 15 (1), 3933. 10.1038/s41598-025-86979-9 39890889 PMC11785764

[B82] Peng W. C. Logan C. Y. Fish M. Anbarchian T. Aguisanda F. Álvarez-Varela A. (2018). Inflammatory Cytokine TNFα promotes the long-term expansion of primary hepatocytes in 3D culture. Cell 175 (6), 1607–1619.e15. 10.1016/j.cell.2018.11.012 30500539 PMC6497386

[B83] Pérez-González C. Ceada G. Greco F. Matejčić M. Gómez-González M. Castro N. (2021). Mechanical compartmentalization of the intestinal organoid enables crypt folding and collective cell migration. Nat. Cell Biol. 23 (7), 745–757. 10.1038/s41556-021-00699-6 34155382 PMC7611697

[B84] Pinkse G. G. M. Bouwman W. P. Jiawan-Lalai R. Terpstra O. T. Bruijn J. A. de Heer E. (2006). Integrin signaling via RGD peptides and Anti-β1 Antibodies Confers resistance to Apoptosis in Islets of Langerhans. Diabetes 55 (2), 312–317. 10.2337/diabetes.55.02.06.db04-0195 16443762

[B85] Raghavan S. Winter P. S. Navia A. W. Williams H. L. DenAdel A. Lowder K. E. (2021). Microenvironment drives cell state, plasticity, and drug response in pancreatic cancer. Cell 184 (25), 6119–6137.e26. 10.1016/j.cell.2021.11.017 34890551 PMC8822455

[B86] Randriamanantsoa S. Papargyriou A. Maurer H. C. Peschke K. Schuster M. Zecchin G. (2022). Spatiotemporal dynamics of self-organized branching in pancreas-derived organoids. Nat. Commun. 13 (1), 5219. 10.1038/s41467-022-32806-y 36064947 PMC9445099

[B87] Recaldin T. Steinacher L. Gjeta B. Harter M. F. Adam L. Kromer K. (2024). Human organoids with an autologous tissue-resident immune compartment. Nature 633 (8028), 165–173. 10.1038/s41586-024-07791-5 39143209 PMC11374719

[B88] Sachs N. Tsukamoto Y. Kujala P. Peters P. J. Clevers H. (2017). Intestinal epithelial organoids fuse to form self-organizing tubes in floating collagen gels. Development 144 (6), 1107–1112. 10.1242/dev.143933 28292848

[B89] Sackett S. D. Tremmel D. M. Ma F. Feeney A. K. Maguire R. M. Brown M. E. (2018). Extracellular matrix scaffold and hydrogel derived from decellularized and delipidized human pancreas. Sci. Rep. 8 (1), 10452. 10.1038/s41598-018-28857-1 29993013 PMC6041318

[B90] Saheli M. Sepantafar M. Pournasr B. Farzaneh Z. Vosough M. Piryaei A. (2018). Three-dimensional liver-derived extracellular matrix hydrogel promotes liver organoids function. J. Cell Biochem. 119 (6), 4320–4333. 10.1002/jcb.26622 29247536

[B91] Saldin L. T. Cramer M. C. Velankar S. S. White L. J. Badylak S. F. (2016). Extracellular matrix hydrogels from decellularized tissues: structure and function. Acta Biomater. 49, 1–15. 10.1016/j.actbio.2016.11.068 27915024 PMC5253110

[B92] Saraswathibhatla A. Indana D. Chaudhuri O. (2023). Cell–extracellular matrix mechanotransduction in 3D. Nat. Rev. Mol. Cell Biol. 24 (7), 495–516. 10.1038/s41580-023-00583-1 36849594 PMC10656994

[B93] Sarker F. A. Prior V. G. Bax S. O’Neill G. M. (2020). Forcing a growth factor response – tissue-stiffness modulation of integrin signaling and crosstalk with growth factor receptors. J. Cell Sci. 133 (23), jcs242461. 10.1242/jcs.242461 33310867

[B94] Sato T. Stange D. E. Ferrante M. Vries R. G. J. van Es J. H. van den Brink S. (2011). Long-term expansion of epithelial organoids from human colon, Adenoma, adenocarcinoma, and Barrett’s Epithelium. Gastroenterology 141 (5), 1762–1772. 10.1053/j.gastro.2011.07.050 21889923

[B95] Sato T. Vries R. G. Snippert H. J. van de Wetering M. Barker N. Stange D. E. (2009). Single Lgr5 stem cells build crypt-villus structures *in vitro* without a mesenchymal niche. Nature 459 (7244), 262–265. 10.1038/nature07935 19329995

[B96] Seino T. Kawasaki S. Shimokawa M. Tamagawa H. Toshimitsu K. Fujii M. (2018). Human pancreatic tumor organoids reveal Loss of stem cell niche factor dependence during disease progression. Cell Stem Cell 22 (3), 454–467.e6. 10.1016/j.stem.2017.12.009 29337182

[B97] Skardal A. Devarasetty M. Rodman C. Atala A. Soker S. (2015). Liver-tumor hybrid organoids for modeling tumor growth and drug response *in vitro* . Ann. Biomed. Eng. 43 (10), 2361–2373. 10.1007/s10439-015-1298-3 25777294 PMC4573342

[B98] Spence J. R. Mayhew C. N. Rankin S. A. Kuhar M. F. Vallance J. E. Tolle K. (2011). Directed differentiation of human pluripotent stem cells into intestinal tissue *in vitro* . Nature 470 (7332), 105–109. 10.1038/nature09691 21151107 PMC3033971

[B99] Stange D. E. Koo B. K. Huch M. Sibbel G. Basak O. Lyubimova A. (2013). Differentiated *Troy*+ Chief cells Act as Reserve stem cells to generate all lineages of the stomach Epithelium. Cell. 155 (2), 357–368. 10.1016/j.cell.2013.09.008 24120136 PMC4094146

[B100] Tejeda-Muñoz N. Morselli M. Moriyama Y. Sheladiya P. Pellegrini M. De Robertis E. M. (2022). Canonical Wnt signaling induces focal adhesion and Integrin beta-1 endocytosis. iScience 25 (4), 104123. 10.1016/j.isci.2022.104123 35402867 PMC8987407

[B101] Tiriac H. Belleau P. Engle D. D. Plenker D. Deschênes A. Somerville T. D. D. (2018). Organoid profiling identifies common Responders to chemotherapy in pancreatic cancer. Cancer Discov. 8 (9), 1112–1129. 10.1158/2159-8290.cd-18-0349 29853643 PMC6125219

[B102] Tong Z. Martyn K. Yang A. Yin X. Mead B. E. Joshi N. (2018). Towards a defined ECM and small molecule based monolayer culture system for the expansion of mouse and human intestinal stem cells. Biomaterials 154, 60–73. 10.1016/j.biomaterials.2017.10.038 29120819 PMC5735007

[B103] Tsai S. McOlash L. Palen K. Johnson B. Duris C. Yang Q. (2018). Development of primary human pancreatic cancer organoids, matched stromal and immune cells and 3D tumor microenvironment models. BMC Cancer 18 (1), 335. 10.1186/s12885-018-4238-4 29587663 PMC5870823

[B104] Valdez J. Cook C. D. Ahrens C. C. Wang A. J. Brown A. Kumar M. (2017). On-demand dissolution of modular, synthetic extracellular matrix reveals local epithelial-stromal communication networks. Biomaterials 130, 90–103. 10.1016/j.biomaterials.2017.03.030 28371736 PMC5461961

[B105] VanDussen K. L. Sonnek N. M. Stappenbeck T. S. (2019). L-WRN conditioned medium for gastrointestinal epithelial stem cell culture shows replicable batch-to-batch activity levels across multiple research teams. Stem Cell Res. 37, 101430. 10.1016/j.scr.2019.101430 30933720 PMC6579736

[B106] Vlachogiannis G. Hedayat S. Vatsiou A. Jamin Y. Fernández-Mateos J. Khan K. (2018). Patient-derived organoids model treatment response of metastatic gastrointestinal cancers. Science 359 (6378), 920–926. 10.1126/science.aao2774 29472484 PMC6112415

[B107] Vukicevic S. Kleinman H. K. Luyten F. P. Roberts A. B. Roche N. S. Reddi A. H. (1992). Identification of multiple active growth factors in basement membrane matrigel suggests caution in interpretation of cellular activity related to extracellular matrix components. Exp. Cell Res. 202 (1), 1–8. 10.1016/0014-4827(92)90397-q 1511725

[B108] Wang L. Stegemann J. P. (2010). Thermogelling chitosan and collagen composite hydrogels initiated with β-glycerophosphate for bone tissue engineering. Biomaterials 31 (14), 3976–3985. 10.1016/j.biomaterials.2010.01.131 20170955 PMC2851195

[B109] Wang W. Jin S. Ye K. (2017). Development of islet organoids from H9 human embryonic stem cells in Biomimetic 3D scaffolds. Stem Cells Dev. 26 (6), 394–404. 10.1089/scd.2016.0115 27960594

[B110] Wang X. Luo Y. Ma Y. Wang P. Yao R. (2024). Converging bioprinting and organoids to better recapitulate the tumor microenvironment. Trends Biotechnol. 42 (5), 648–663. 10.1016/j.tibtech.2023.11.006 38071145

[B111] Wang Y. Lee J. H. Shirahama H. Seo J. Glenn J. S. Cho N. J. (2016). Extracellular matrix functionalization and Huh-7.5 cell Coculture promote the hepatic differentiation of human adipose-derived mesenchymal stem cells in a 3D ICC hydrogel scaffold. ACS Biomater. Sci. Eng. 2 (12), 2255–2265. 10.1021/acsbiomaterials.6b00487 33465898

[B112] Yanai I. Haas S. Lippert C. Kretzmer H. (2024). Cellular atlases are unlocking the mysteries of the human body. Nature 635 (8039), 553–555. 10.1038/d41586-024-03552-6 39567780

[B113] Yang L. Wang X. Zhou X. Chen H. Song S. Deng L. (2025). A tunable human intestinal organoid system achieves controlled balance between self-renewal and differentiation. Nat. Commun. 16 (1), 315. 10.1038/s41467-024-55567-2 39747097 PMC11697020

[B114] Yoon Y. J. Kim D. Tak K. Y. Hwang S. Kim J. Sim N. S. (2022). Salivary gland organoid culture maintains distinct glandular properties of murine and human major salivary glands. Nat. Commun. 13 (1), 3291. 10.1038/s41467-022-30934-z 35672412 PMC9174290

[B115] Zhang Y. S. Aleman J. Shin S. R. Khademhosseini A. Kim D. Mousavi Shaegh S. A. (2017). Multisensor-integrated organs-on-chips platform for automated and continual *in situ* monitoring of organoid behaviors. Proc. Natl. Acad. Sci. 114 (12), E2293–E2302. 10.1073/pnas.1612906114 28265064 PMC5373350

[B116] Zhao Y. Li S. Zhu L. Huang M. Xie Y. Song X. (2024). Personalized drug screening using patient-derived organoid and its clinical relevance in gastric cancer. Cell Rep. Med. 5 (7), 101627. 10.1016/j.xcrm.2024.101627 38964315 PMC11293329

